# A Comparison of Waste Stability Indices for Mechanical–Biological Waste Treatment and Composting Plants

**DOI:** 10.3390/ijerph15112585

**Published:** 2018-11-19

**Authors:** Andrzej Jędrczak, Monika Suchowska-Kisielewicz

**Affiliations:** Institute of Environmental Engineering, University of Zielona Góra, 65-417 Zielona Góra, Poland; a.jedrczak@iis.uz.zgora.pl

**Keywords:** MBT, respirometric activity (AT4), biogas potential (GB21)

## Abstract

Achieving high efficiency of biological waste treatment in mechanical–biological treatment (MBT) plants requires reliable methods for measuring the degree of biodegradation of organic substances. For this purpose, several physical, chemical, and biological indices are used. This paper presents respirometric activity (AT4), biogas potential (GB21), total and dissolved organic carbon (TOC and DOC, respectively), and loss on ignition (LOI) values, as well as the correlations between the indices selected for stabilized waste produced in 18 MBT plants in Poland, which use various technologies for biological processing of the organic fraction of municipal solid waste. The study confirms that there is a linear relationship between AT4 and GB21 for stabilized waste produced in MBT plants, regardless of the waste treatment technology used. It has also been found that there is a linear relationship between AT4 and the concentration of dissolved carbon in water extract from stabilized waste. This indicates that DOC can be used for monitoring the organic matter stabilization process in mechanical–biological waste treatment plants. Its advantage is a shorter time needed for measurements in comparison to AT4 and GB21 tests.

## 1. Introduction

The main objective of the biological process that municipal solid waste (MSW) goes through in mechanical–biological treatment plants (MBT) is the stabilization of biodegradable organic fractions. The purposes of the stabilization are (i) volume reduction, (ii) minimization of structural changes occurring in a landfill, (iii) reduction of biogas production, and (iv) susceptibility to washing out or settling after being deposited. Analysis of the efficiency of waste treatment in MBT plants requires reliable methods for measuring the degree of biodegradation of organic substances. This parameter not only provides valuable information for landfill operators on the acceptability of substrates for final disposal, but also makes it possible to assess the efficiency of operating plants, as well as improving biological treatment, designing optimized plants, and determining the potential environmental impact of stabilized waste produced in them.

Several physical, chemical, and biological indices have been proposed for measuring the degree of waste stabilization.

The non-biological methods used for monitoring the course of biological processes include such parameters as the content of organic matter (LOI), total and dissolved organic carbon (TOC and DOC, respectively), chemical oxygen demand (COD), and C/N [1,2,3,4]. The methods available allow for relatively quick and inexpensive quantification of these parameters. LOI, TOC, and DOC are indicators that have traditionally been used to determine the content of organic matter in waste [5]. Nowadays, also for waste characterisation, the COD parameter—an indicator traditionally used to assess the content of organic pollutants in waste water and sewage sludge—is also used. However, the results of indexing MSW, which is a very heterogeneous material, will only partly predict its subsequent behaviour and they are subject to significant error due to the presence of non-biodegradable, volatile matter or substances susceptible to oxidation. Therefore, in addition to traditional indicators, biological indicators that better reflect the amount of biodegradable (reactive) organic carbon contained in processed waste and the correlation between them, as well as physical and chemical indicators, have been studied.

Biological methods represent two groups of tests: aerobic (respirometric) (AT4, DRI) and anaerobic (methanogenic activity) (GB21) [2,6,7,8,9,10,11,12]. These include the following:

- Anaerobic tests
  - BMP: biochemical methane potential, with a test duration of up to 100 days,
  - GS21: total gas production, with a test duration of 21 days,
  - GB21: biogas production evaluation, with a test duration of 21 days,
- Respirometry tests
  - AT4: static respiratory test, with a test duration of 4 days,
  - DR4 or DR100: dynamic respiratory test, with a test duration of 4 and 100 days, respectively.

Particular tests differ in terms of the basic parameters of the testing process, such as temperature, duration, and the mass of the sample. Some of these have been adopted for assessing the degree of decomposition of biodegradable wastes in MBT processes by the legislation of some European countries, e.g., Austria (BGBl. No. II 39/2008), Germany [13], Italy [8], England, and Wales [14]. According to numerous publications, there is a good correlation between various biological indices [15,16,17,18,19]. However, determining these indices is problematic for a number of reasons, such as the long duration of tests, high costs of respirometers, and possible understatement of the measured parameters caused by toxic substances present in the waste, which may have a negative impact on the activity of microorganisms during the measurement process.

Cossu and Raga [16] and Cossu et al. [19,20] proposed to measure the ratio of biological oxygen demand in five days/chemical oxygen demand (BOD_5_/COD) in water extract from waste as a more appropriate parameter than traditional respirometric indices and biogas production measured directly in solid samples. They also found a positive correlation between the BOD_5_/COD parameter and the AT4 index. This method is simple, and requires only standard equipment available in company laboratories. In further studies, the authors will determine the reference conditions for indexing [20].

In addition to numerous publications on the correlation between biological indicators AT4 and GB21 for MBT products [14,15,16,17,18,19], there is a lack of widely documented data on the correlation between biological indicators (AT4 and GB21) and non-biological indicators (DOC and ΔLOI). The aim of the study was to determine these relationships for the products of MBT plants that use different waste treatment technologies (aerobic and anaerobic) in different reactor types and under different conditions. The conclusion that there are significant relations between the examined indicators will open the possibility of using non-biological indicators to monitor the stabilization process of the organic fraction of municipal solid waste (OFMSW) in mechanical–biological waste treatment plants. The advantage of these non-biological indicators is the shorter time needed for measurements compared to AT4 and GB21 tests.

The paper presents AT4, GB21, and DOC values, the organic fraction decomposition achieved, expressed as a reduction in the loss on ignition (LOI), total organic carbon (TOC), and TDS (total dissolved solids), as well as the correlations between the indices selected (biological indicators (AT4 and GB21) and non-biological indicators (DOC and ΔLOI) for stabilized waste produced in 18 MBT installations in Poland, which use various technologies for biological processing of the organic fraction of MSW.

LOI is often used, as it is the simplest and cheapest method for determining the content of organic matter in waste. Many authors have studied the relationship between this parameter and BMP. The obtained correlations assume different values of the correlation coefficient (R2) from 0.17 to 0.77, depending on the type of waste tested and the method of its processing [5]. According to some of the literature data, DOC also shows a constant tendency to decrease in value depending on the degree of waste stabilization, and shows a good linear correlation between DOC and BMP (R2 = 0.97) [21]. The data presented in this paper supplements knowledge on the correlation between biological and non-biological indices used to assess the biodegradation susceptibility of waste before and after the biological stabilization process carried out in MBT plants. In professional literature there is no widely documented data on these correlations determined for MBT plants that use different waste treatment technologies (aerobic and anaerobic) for waste treated in different types of reactors and under different conditions.

## 2. Materials and Methods

### 2.1. Waste Samples

The waste samples used in this study consisted of the organic fraction of municipal solid waste (OFMSW) that was produced in a full-scale MBT plant (the undersized MSW fraction from an 80 mm trammel) and directed for biological treatment, and the final product of this treatment is called stabilized waste (SW). Representative samples came from full-size plants that used different waste treatment technologies (Table 1).

The general OFMSW and SW samples were prepared by collecting 10 primary samples with a minimum mass of 10 kg each from the places where they had been produced at the plants at regular time intervals, or from different sites of the windrow (in the case of SW). The general samples obtained in this way were divided into parts made up of over 20 kg (OFMSW) or over 10 kg (SW). The OFMSW samples were divided into two equal samples. In the first sample, the material composition was determined, whereas the second sample and the stabilized waste samples were transported immediately to the laboratory, where they were kept for a maximum period of 24 h at 4 °C to impede biological activity. In each plant, three final OFMSW samples and three stabilized waste samples were collected.

The samples were collected between January and May 2015. The OFMSW and SW samples intended for testing came from the same batch of processed waste.

The samples were appropriately homogenised in the laboratory and subsequently used to prepare representative test samples. Test samples for biological tests and leachate analysis were prepared from fresh samples, which had undergone size reduction to a final size of under 10 mm.

### 2.2. Scope of Study

In the solid, non-pretreated samples, the following parameters were determined (each determination was duplicated):

○ OFMSW: material analysis of the fraction, moisture level, loss on ignition, and organic carbon
○ SW: moisture level, loss on ignition, organic carbon, AT4, and GB21.

DOC and TDS (total dissolved solids) were not determined from the samples as such, but from well-defined water extracts taken from the SW.

The material and physico-chemical analysis of the wastes was carried out according to the standards and procedures presented below:

1. Material analysis of the OFMSW sample. The scope of material analysis included the following components: organic, paper, plastics, glass, textiles, metals, and inert others (PN-Z-15006: 1993).
2. Determination of moisture content in the OFMSW samples and SW–PN-Z-15008/02: 1993, as well as PN-EN 14346: 2011.
3. Determination of the loss on ignition (LOI) for the OFMSW samples and SW–PN-EN 15169: 2011+Ap1:2012
4. Determination of total organic carbon (TOC) for the OFMSW and SW–PN-EN 13137: 2004
5. Determination of AT4 in the SW—demand for oxygen (AT4)–PB-06 of 07.10.2014, edition 2 (Point 2.2.1)
6. Determination of GB21 in the SW—determination of GB21 (Point 2.2.1)
7. Preparation of water extract from the SW—preparation of water extract PB-05 z of 13.03.2012, edition 1, PN-Z-15009: 1997 (Point 2.2.3)
8. Determination of dissolved organic carbon (DOC): PN-EN 1484: 1999
9. Determination of total dissolved solids (TDS): PN-EN 12457-4: 2006

#### 2.2.1. Determination of the Demand for Oxygen for the Biochemical Decomposition of Biodegradable Fractions within Four Days

AT4 was measured for waste samples without glass, stones, and metals; the samples were hand-crushed and sieved through a 10 mm sieve with a moisture content of 40–50%, at a temperature of 20 ± 1 °C. The mass of the mineral components was taken into account in the calculations. The demand for oxygen was measured over four days after the adaptation stage (lag stage) in OxiTop devices. The standard requires that this stage should end when the average demand for oxygen during 3 h reaches 25% of the 3-h average value that occurs during the maximum increase in oxygen consumption during the first four days. The mass of oxygen consumed during the lag stage was not included in the mass of oxygen consumed during the whole test (lag stage + four days). The mass of oxygen from the lag stage cannot exceed 10% of the total demand for oxygen during the first four days.

#### 2.2.2. Determination of Biogas Potential in the Stabilized Waste

GB21 was determined in a laboratory in a 30-station fermenter for periodic fermentation. Each station consisted of three elements, forming a digester chamber: a 1 L cylinder; a gas burette with a capacity of 2.30 dm^3^, for measuring the amount of biogas produced; and a bottle with a saturated sodium chloride solution for pressure equalization.

The thermostat was a metal tub filled with water heated in the reactors to the required temperature—i.e., 36 °C for mesophilic fermentation. Water circulation pumps and two contact thermometers were installed in the tub. These were connected to a control device that controlled the operation of the heaters (turned them on or off) based on thermometer indications.

A prepared waste sample of 100 g with a known moisture content was placed in the reactor. A sample of 0.100 dm^3^ of properly prepared seed material (fermented sewage sludge) was added to the reactor. Distilled water was added to the material in the reactor to a volume of 0.600 dm^3^, the pH of the prepared mixture was adjusted (to a pH range of 6.8–7.8), and the reactor was tightly closed with a rubber stopper. The air was removed from the space above the surface of the mixture in the reactor by blowing nitrogen through it for five minutes to create anaerobic conditions. The reactor was then tightly connected to gas burettes and placed in a thermostat. The endogenous activity of the seeding material (control samples without wastes) was determined in separate reactors.

At least once a day (or more often if necessary) the methane fermentation process was checked by measuring the volume of the biogas produced, as well as the air pressure and air temperature in the room. The study was carried out for 21 days, beginning from the end of the lag phase.

It was assumed that the lag phase ended on the day when the average daily gas production reached 25% of the maximum three-day average calculated over the test period. The gas volume generated during the lag stage was not included into the gas volume generated during the 21-day test period. The volume of biogas was adjusted to the standard temperature and pressure conditions. The result was presented in N dm^3^kg^−1^ DM (dry matter).

The composition of biogas was determined periodically when enough biogas had accumulated in the burette for proper composition analysis. The content of methane and carbon dioxide (also of hydrogen sulphide and ammonia) was determined in the biogas. This was done with a Geotechnical Instruments GA2000 Plus device.

#### 2.2.3. Preparation of Water Extract from the Stabilized Waste

A waste sample of about 40 ± 0.5 g DM reduced in size to grains of <10 mm was placed in a HDPE bottle and filled with 400 mL of redistilled water (the water to dry weight ratio of the sample was 10/1), in accordance with PN-Z-15009: 1997. After 1 h, the bottle was closed with a stopper and shaken for 4 h. The process of elution was carried out at room temperature. The bottle was then opened and left for 16 h, protected against light. After that, the bottle with the sample was shaken again for 4 h and left for 2 h for the solids to sediment; then the liquid was centrifuged and collected in a labelled conical flask.

The following were determined in the extracts: dissolved organic carbon (DOC), using a Shimadzu TOC-V CSN analyser; and the content of total dissolved solids (TDS).

## 3. Results and Discussion

### 3.1. Organic Fraction of Municipal Solid Waste Characteristics

**Figure 1:**
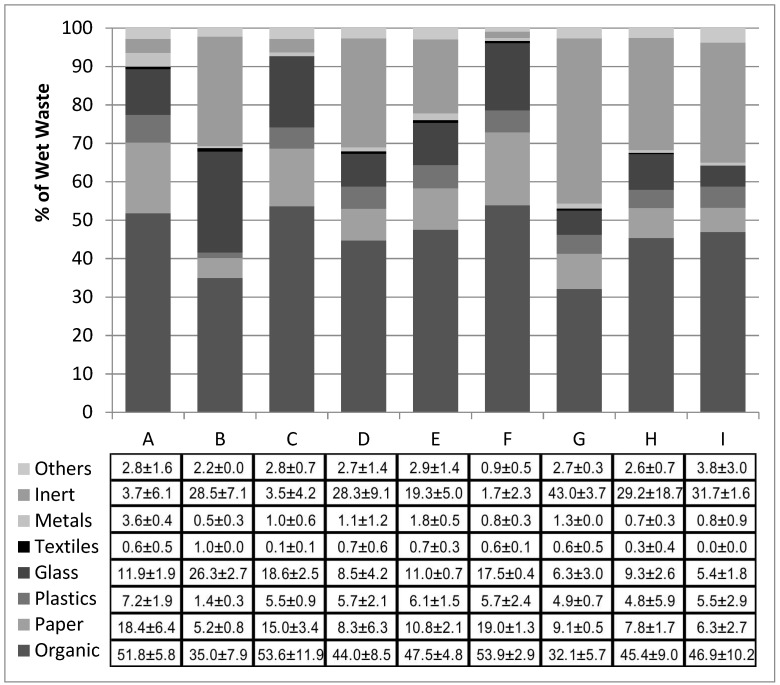
The average composition of the organic fraction of municipal solid waste (OFMSW) samples directed for biological treatment in the plants that used particular waste treatment technologies.

Figure 1 presents the average composition of the OFMSW samples directed for biological processing in the plants used in the tests, which is expressed as the percentage of the total wet mass. Table 2 presents the average content of moisture, organic matter (loss on ignition), and organic carbon (C_org_) in the samples.

The average composition of the OFMSW directed for biological treatment in the plants used in the study was varied. The lowest organic content was found in the OFMSW directed for stabilization to containers, where column G reached 32.1%, as illustrated in Figure 1, and the OFMSW for thermophilic fermentation, where column B reached 35%. The organic content share in the other wastes ranged from 42.5 to 53.9%.

The OFMSW contained on average 39.6 ± 12.5% of water. All waste directed for biostabilization in aerobic conditions required hydration outside installation F. The OFMSW placed in steel containers in this installation had the highest moisture content—60.6%. The lowest moisture content (24.6%) was found in the OFMSW directed for stabilization in outdoor windrows (G).

Assessment of the results presented in Table 2, based on analysis of variance (ANOVA), indicates that there are no significant differences between the values of organic carbon (LOI, TOC) found in the stabilized waste in particular technological plants; however, there are significant differences in the moisture content (NIR = 27.7).

**Figure 2:**
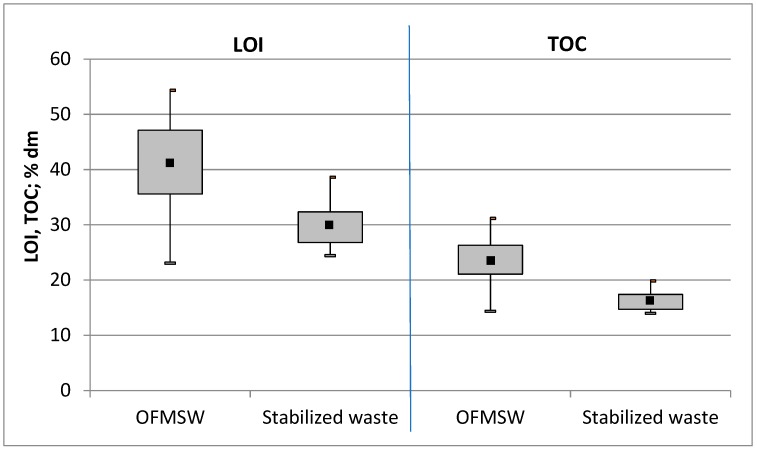
The range of the loss on ignition (LOI) and total organic carbon (TOC) parameters (% DM (dry matter)) obtained for 20 different waste samples from Polish mechanical–biological treatment (MBT) plants. The diagram includes the median, minimum, and maximum values, as well as the 25% and 75% quartiles.

Figure 2 presents the ranges of LOI and TOC values for the OFSMW directed for treatment and for the SW produced in 18 MBT installations. Volatile matter was present in the SW, i.e., the final product after biological stabilization, which is intended for storage in a landfill, in an amount of 24.4 to 38.6% DM, with a median of 29.8% DM. The wide range of values can be attributed to the highly varied composition of the OFMSW and the different biological processing systems in the installations used in this study. The median of these parameters for the SW fell to 28% below the value determined for the OFMSW. TOC was present in the SW in an amount of 14.0 to 19.9% DM, with a median of 16.1% DM. The median for the SW fell to 32% below the value determined for the OFMSW, and it represents the efficiency of the biological waste treatment. The remaining content of TOC in the SW was about 16% DM (median).

**Figure 3:**
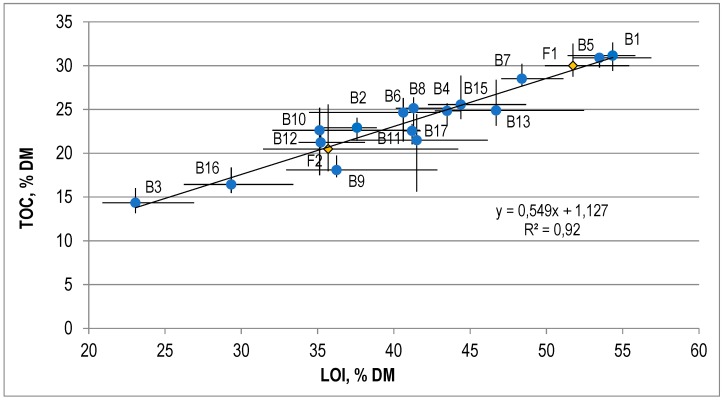
The correlation between the loss on ignition and the content of organic carbon in the OFMSW.

The average value of the loss on ignition was 41.1 ± 8.2% DM, and the average organic carbon content was 23.7 ± 4.7% DM. There was a linear relationship between the values of these indices (Figure 3). The coefficient of correlation (R2) for this relationship was high, and reached 0.92.

### 3.2. Correlation between Biogas Potential and Respirometric Activity for the Stabilized Waste

The AT4 values for the SW were within the range of 1.3 to 17.1 mg O_2_gDM^−1^. The German ordinance on the storage of MSW [13] specifies the following criteria for acceptance of wastes at landfills after mechanical and biological treatment: an AT4 value below 5 mg O_2_gDM^−1^ or GB21 below 20 dm^3^Lkg^−1^ DM. The Austrian ordinance on landfills specifies the following requirements for waste storage after MBT: AT4 below 7 mg O_2_gDM^−1^ or GB21 below 20 dm^3^kg^−1^ DM. In Poland, it is recommended to continue the process of biological treatment until an AT4 value below 10 mg O_2_ gDM^−^^1^ is reached [22]. AT4 values exceeding 10 mg O_2_gDM^−1^ were found in five SW samples, and values higher than 5 mg O_2_g^−1^DM in 14 SW samples. The main reason for the high AT4 values in some of the SW samples was an insufficient amount of water added to the OFMSW during the treatment stage (after the stage of intensive biostabilization, the moisture content in the wastes fell below 30% in two of the plants, installations B9 and B13, which was unacceptable) and the treatment time, which was too short. An eight-week treatment period seems insufficient to achieve the required level of waste stabilization in plants where waste is shovelled only once (plants B5, B6, and B17).

The production of biogas (GB21) ranged from 0.4 to 47.8 NL kgDM^−1^, and the production of methane from 0.3 to 24.9 NL kgDM^−1^. The average content of methane in biogas was about 60% (*v*/*v*).

The results of the study confirm the existence of a linear relationship between AT4 and biogas production, which was mentioned in earlier publications [15,16,17,18,19].

**Figure 4:**
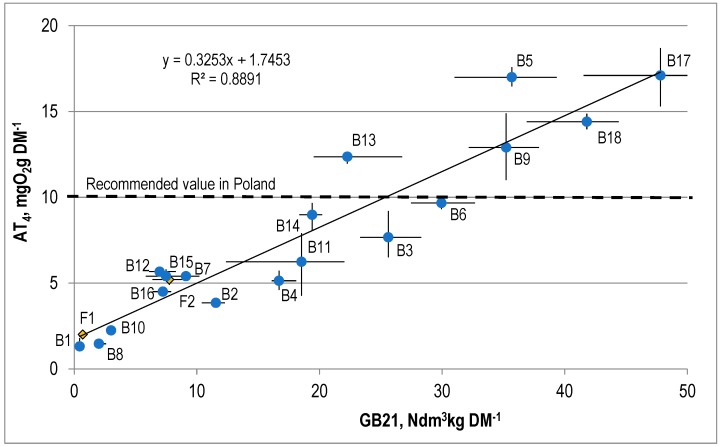
The correlation between AT4 and biogas potential (GB21) for the stabilized waste.

This relationship is described by the following equation: AT4 = 0.325·GB21 + 1.745 (Figure 4). The coefficient of correlation (R2) for this correlation was 0.89. It was observed that the waste treatment technology had not had any impact on the above correlation.

In addition, Bohrn et al. [23] showed a good correlation between AT4 and BMP for wastes processed in 13 installations with various MBT technologies, obtaining a correlation coefficient of 0.89 for the representative samples of wastes selected from a total of 319 samples. Cossu et al. [16] and Ponsa et al. [17] set the correlation coefficient between these parameters in the range of 0.63 to 0.94.

Previous research by Fricke et al. [24] shows that waste after biological oxygen stabilization, or a combination of anaerobic and aerobic stabilization for the average AT4 value (5 mgO_2_Gdm^−1^), obtains an average GB21 value of 12–15 dm^3^kgDM^−1^. The regression curve determined by them also indicates that AT4 values of about 1 mgO_2_gDM^−1^ correspond to the value of GB21 of about 0 dm^3^kgDM^−1^. This is consistent with the general observed relationship showing that some of the organic substances decomposable under aerobic conditions are not available to decompose in anaerobic processes, e.g., lignin.

A similar tendency was observed in the present studies—AT4 values at the level of 5 mgO_2_g DM^−1^ correspond to the value of GB21 of 10 Ndm^3^kgDM^−1^, and the GB21 value of about 0 dm^3^kgDM^−1^ corresponds to the AT4 value of approximately 1.7 mg O_2_gDM^−1^ (Figure 4).

### 3.3. Correlation between the Reduction of Volatile Matter, Organic Carbon, Dissolved Organic Carbon, and Respirometric Activity

**Figure 5:**
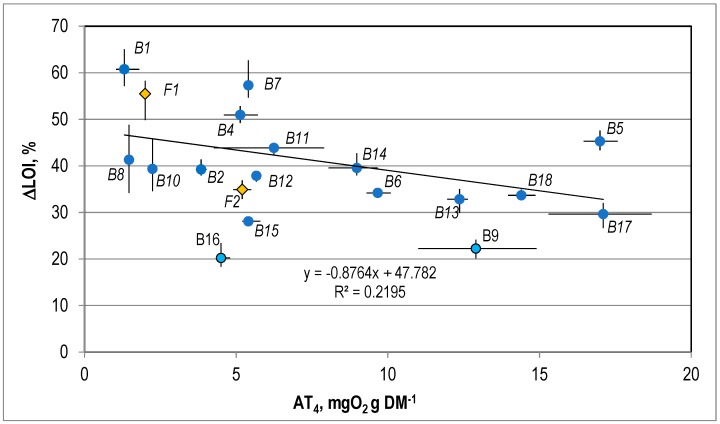
The correlation between the LOI and the AT4 for the stabilized waste.

**Figure 6:**
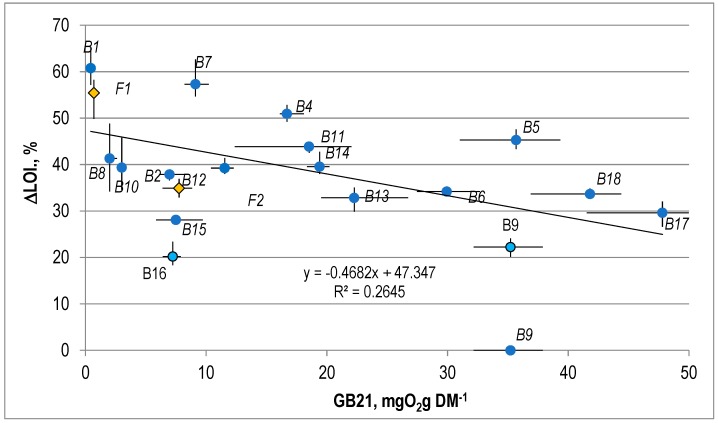
The correlation between the LOI and the GB21 for the stabilized waste.

The correlation between the reduction of LOI in the biological treatment process, AT4, and GB21 is presented in Figure 5 and Figure 6.

**Figure 7:**
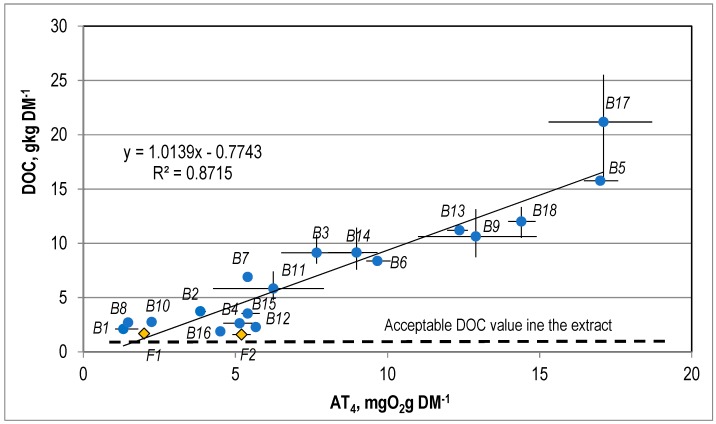
The correlation between DOC and AT4 for the stabilized waste.

**Figure 8:**
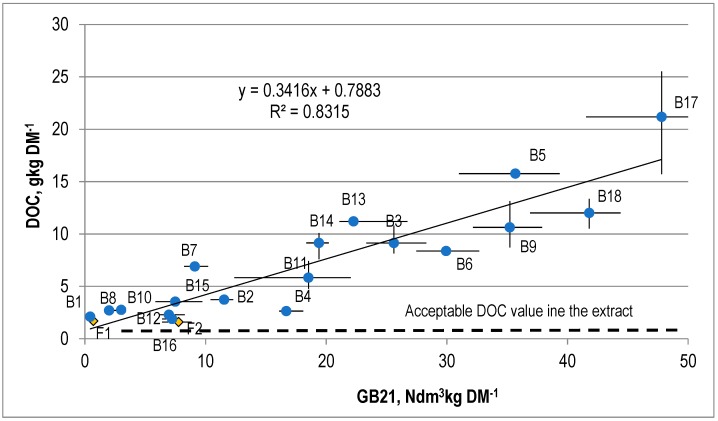
The correlation between DOC and GB21 for the stabilized waste.

The correlation between DOC in the water extract from the stabilized waste and AT4 and GB21 is presented in Figure 7 and Figure 8.

The reduction of the loss on ignition (ΔLOI) was calculated on the basis of the LOI of the waste directed for biological treatment (LOI_O_) and of the stabilized waste (LOI_S_), according to the equation (Paredes 2000):

(1) $$\Delta_{\mathrm{LOI}}=100-100\left[\frac{{\mathrm{LOI}}_{S}\cdot \left(100-{\mathrm{LOI}}_{O}\right)}{{\mathrm{LOI}}_{O}\cdot \left(100-{\mathrm{LOI}}_{S}\right)}\right]$$

The values of the reduction of the loss on ignition during the biological treatment were within the range of 28.1 to 60.7%, with a median of 39.4%, excluding the samples obtained in plants B9 and B16. In plants B9 and B16, the values for the reduction of the LOI were 22.2% and 20.2%, respectively.

It was found that there was no linear correlation between ∆LOI and the value of AT4 (Figure 5) or GB21 (Figure 6) for the stabilized waste. The values of the coefficient of correlation (R2) for these correlations were 0.22 and 0.21.

The water extracts from the SW subjected to analysis were highly saline and contained very large amounts of dissolved organic compounds. The TDS values were within the range of 21.3 to 86.5 g kgDM^−1^ (on average 44.8 ± 15.5 g kgDM^−1^), and the acceptable value for wastes sent to landfills is 60 g kgDM^−1^. The content of dissolved organic carbon in the SW was from 1.6 up to 21.2 g kgDM^−1^ (an average of 6.6 ± 4.1 g kgDM^−1^), and the acceptable value is 800 mg kgDM^−1^.

There is a linear correlation between the DOC values for the SW and the AT4 and GB21, which is described by the equations DOC = 1.014·AT4 − 0.774 (Figure 7) and DOC = 0.3416·GB21 − 0.7883 (Figure 8). The coefficient of correlation (R2) for this correlation is 0.87 and 0.83.

Poganani et al. [25] showed that an interesting indicator for assessing the degree of stabilization of waste is the quotient of total organic carbon to biodegradable organic carbon (BOD), where BOD is determined based on the CO_2_ content in biogas. The quotient is characterized by a high correlation between AT4 and GB21 with the determination coefficient R2 = 0.88. Cossu and Raga [16] and Cossu et al. [19,20] proposed to measure the biological oxygen demand over five days/chemical oxygen demand (BOD_5_/COD), claiming it to be a stable indicator that is not significantly affected by the presence of impurities in the sample.

Further research is necessary, however, to assess the suitability of these indicators to determine the degree of stabilization of waste treated in MBP installations under various technical and technological conditions.

## 4. Conclusions

Different waste treatment technologies are used in MBT plants in Poland. They differ in terms of the duration and configuration of the intensive decomposition and maturation stage, and also in terms of the method and frequency of waste shovelling. The composition of wastes is also different. This diversity results in a wide range of values of parameters.

The total duration of OFMSW biostabilization ranged from 42 to 135 days, including the intensive phase carried out in closed reactors for 18 to 84 days, and the phase of maturation in windrows, which lasted for 13 to 63 days. This diversifies results across a wide range of parameter values. AT4 values for stabilized waste ranged from 1.3 to 17.1 mgO_2_gDM^−1^, and GB21 from 0.4 to 47.8 Ndm^3^kgDM^−1^.

In spite of this, the study confirms that there is a linear correlation between AT4 and biogas production for the SW produced in MBT plants, regardless of the waste treatment technology used. There is also a linear correlation between AT4 and the concentration of dissolved carbon in the water extract from the SW. This parameter can be used for monitoring the organic matter stabilization process in mechanical–biological waste treatment plants. Its advantage is a shorter duration in comparison to AT4 and GB21 tests. No correlation was found between the reduction of volatile matter and organic carbon in the process of biological treatment of the OFMSW and the AT4 value of the SW.

The results of the study also indicate that an eight-week period of biostabilization in aerobic conditions seems insufficient to effectively reduce the reactivity of the OFMSW.

## Figures and Tables

**Table 1:** The plans and waste samples examined in this study.

System	Samples	Intensive Phase of Biostabilization		Maturation in Windrows, Days
		Reactor	Duration Time, Days	
A	F1	Mesophilic dry fermentation of fraction <60 mm, after removal of Fe and hard particles	25	20
B	F2	Thermophilic dry fermentation of fraction <40 mm without Fe and 40–80 mm after removal of Fe and hard particles	26	21
C	B1	Reinforced concrete cells in the hall, with forced aeration and waste transfer	24	42
	B2		28	33
	B3		25	41
D	B4	Reinforced concrete reactors with reinforced concrete or plastic roofs	29	42
	B5		35	24
	B6		24	33
	B7		23	19
	B8		29	55
	B9		27	41
E	B10	Cells with gates and roofs covered with GORE^®^ laminates	30	47
	B11		22	63
F	B12	Steel reactors (containers)	18	35
G	B13, B14	No reactor, the whole process is carried out in windrows in open area	-	84, 134
H	B15	Foil sleeves	51	-
	B16		50	
I	B17, B18	Windrows covered with GORE^®^ laminates	56, 70	-

**Table 2:** The average values of the moisture content, organic content (LOI), and total organic carbon (TOC) in the OFMSW samples directed for biological treatment in the plants that used particular waste treatment technologies.

Determinations	Average Values for Installations Using Particular Waste Treatment Technologies								
	A	B	C	D	E	F	G	H	I
Moisture, %	53.5 ± 1.4	34.7 ± 2.6	33.9 ± 10.5	41.8 ± 12.3	39.6 ± 6.9	60.6 ± 6.3	24.6 ± 1.4	34.5 ± 12.4	38.8 ± 6.2
Loss on ignition, % DM	51.7 ± 3.0	35.7 ± 6.9	38.3 ± 14.2	43.9 ± 7.6	38.2 ± 7.9	35.2 ± 2.5	46.7 ± 5.0	36.9 ± 8.9	41.5 ± 7.0
Total organic carbon, % DM	30.0 ± 2.1	20.5 ± 4.2	22.8 ± 7.4	25.4 ± 12.0	22.6 ± 2.9	21.2 ± 1.1	24.9 ± 3.0	21.0 ± 5.3	21.5 ± 4.6
